# A novel technique for large colorectal specimen retrieval after endoscopic submucosal dissection: anoscope-assisted tumor extraction by defecation

**DOI:** 10.1055/a-2673-9129

**Published:** 2025-08-14

**Authors:** Stefano Kayali, Stefano Andreotti, Giorgio Nervi, Luigi Laghi

**Affiliations:** 1 9370 Department of Medicine and Surgery, University of Parma, Parma, Italy; 2 18630 Gastroenterology and Endoscopy Unit, University Hospital of Parma, Parma, Italy

Colorectal endoscopic submucosal dissection (ESD) has emerged as the gold standard for treating extensive superficial colorectal neoplasms, allowing the en bloc excision of particularly large lesions 1 2 . Preserving the integrity of the specimen ensures an optimal histopathological evaluation can be performed. Lesions up to 6 cm are typically retrieved using a basket net; with larger specimens, traditional devices often prove insufficient, making extraction without damage challenging.

To address this challenge, various research groups have developed innovative techniques. Among these, Fukita et al. 3 proposed using an anoscope to facilitate specimen extraction, after it had been grasped using a retrieval net. Another notable method is the tumor extraction by defecation (TED) technique 4 , where the specimen is expelled transanally via the Valsalva maneuver performed by the patient. In the first approach, the techniqueʼs effectiveness is limited by the size of the retrieval net, as an incomplete grasp may result in specimen damage. In the second method, the patientʼs ability to generate sufficient contractile force for specimen expulsion may be impaired, particularly following prolonged sedation.

We have developed a novel and efficient technique for retrieving large specimens, namely the anoscope-assisted tumor extraction by defecation (AA-TED) method, which combines the benefits of the Valsalva maneuver and anoscope-assisted retrieval ( Video 1 ). At the end of the resection procedure, an anoscope is inserted into the rectum, followed by reinsertion of the endoscope and saline irrigation. The patient then performs the Valsalva maneuver. The resulting increase in intra-abdominal pressure, along with the anoscope-induced reduction in resistance of the internal and external anal sphincters, enables passage of the specimen through the anal canal ( Fig. 1 ).

Removal of a large colorectal lesion resected by endoscopic submucosal dissection (ESD) using the anoscope-assisted tumor extraction by defecation (AA-TED) technique showing lubrication and insertion of the anoscope, the specimen in the anoscope during the Valsalva maneuver, and its subsequent extraction.Video 1

**Fig. 1:**
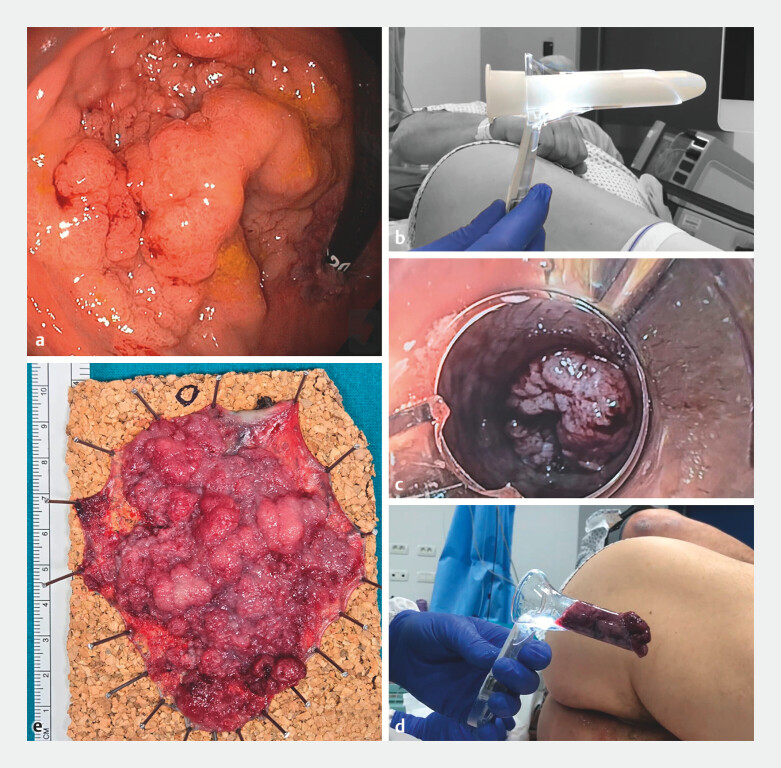
Images from the removal of a large colorectal lesion by endoscopic submucosal dissection (ESD) using the anoscope-assisted tumor extraction by defecation (AA-TED) technique showing: **a** a laterally spreading tumor of granular nodular mixed type in the lower rectum involving the dentate line; **b** photograph of the anoscope; **c** appearance of the lesion in the anoscope during the Valsalva maneuver; **d** the extracted specimen in the anoscope; **e** macroscopic appearance of the resected specimen.

In two consecutive cases involving large lesions (mean maximum diameter of 88 mm), we successfully employed this technique after the failure of both conventional retrieval nets and the TED technique. Based on this experience, we consider AA-TED to be a useful method for retrieving large colorectal lesions following ESD.

Endoscopy_UCTN_Code_TTT_1AQ_2AF

## Notes

Endoscopy E-Videos https://eref.thieme.de/e-videos
*E-Videos* is an open access online section of the journal *Endoscopy* , reporting on interesting cases and new techniques in gastroenterological endoscopy. All papers include a high-quality video and are published with a Creative Commons CC-BY license. Endoscopy E-Videos qualify for HINARI discounts and waivers and eligibility is automatically checked during the submission process. We grant 100% waivers to articles whose corresponding authors are based in Group A countries and 50% waivers to those who are based in Group B countries as classified by Research4Life (see: https://www.research4life.org/access/eligibility/ ). This section has its own submission website at https://mc.manuscriptcentral.com/e-videos .
